# An exploration into the challenges of managing the COVID-19 pandemic in mass education centers in Iran: A qualitative content analysis

**DOI:** 10.34172/hpp.2023.08

**Published:** 2023-04-30

**Authors:** Hosein Mahmoudi, Fakhrudin Faizi, Abolfazl Rahimi, Shirdel Zandi

**Affiliations:** ^1^Trauma Research Center, Nursing Faculty, Baqiyatallah University of Medical Sciences, Tehran, Iran; ^2^Atherosclerosis Research Center and Nursing Faculty, Baqiyatallah University of Medical Sciences, Tehran, Iran; ^3^Nursing Faculty, Baqiyatallah University of Medical Sciences, Tehran, Iran; ^4^School of Nursing, Baqiyatallah University of Medical Sciences, Tehran, Iran

**Keywords:** COVID-19, Challenges, Educational centers, Pandemics, Disease management

## Abstract

**Background:** During the COVID-19 pandemic, Iran’s mass education centers, which house a large number of trainees, encountered numerous difficulties in managing the disease. Understanding these challenges can help manage future pandemics. This study was conducted to explore the challenges of managing the COVID-19 pandemic in mass education centers in Iran.

**Methods:** In this qualitative study, we used a qualitative content analysis of data collected from June to October 2022 in eight mass education centers in Iran. Semi-structured interviews (n=19) were used for data collection.

**Results:** Four main themes and eleven subthemes were identified: The essence of dormitory life (Subthemes included: "The high population density in the dormitory", "Public toilets" and, Interprovincial travel), the inflexibility of the profession (Subthemes included: "Inapplicable health protocols" and, "Inflexible rules and regulations"), Negligence (Subthemes included: "Not adhering to health protocols", "Non acceptance of illness", and "High-risk taking"), and Weakness of health-care platform (Subthemes included: "shortage of healthcare facilities", "Lack of specialized personnel", and "The uni-dimensional aspect of healthcare services").

**Conclusion:** We identified several challenges that made the handling of COVID-19 difficult in Iran’s centers for mass education. These findings can help future research in addressing the challenges and designing adaptable plans for pandemic management in mass education centers.

## Introduction

 Throughout history, pandemics have been regarded as one of the biophysical risks that pose a threat to human societies.^1^ One of the most critical and dangerous pandemics in human history was identified in Wuhan, China, in December 2019 and spread rapidly.^2^ This pandemic is a contagious respiratory disease caused by acute upper respiratory syndrome coronavirus-2 (SARS-CoV-2).^3^ This disease quickly spread worldwide, so on March 11, 2020, the World Health Organization (WHO) classified COVID-19 as a pandemic.^4^

 COVID-19 has become a challenging problem for organizations, countries, and the world. It disrupted the routine performance of many vital organizations in every country and caused a big shock to the global economy and trade.^5,6^

 Education was one of the most critical activities disrupted worldwide after the spread of COVID-19. In fact, the COVID-19 pandemic created the most extensive disruption of education systems in human history, affecting nearly 1.6 billion learners worldwide. The shutdown of schools, institutions, and other learning environments has disrupted the education of almost 94% of the world’s students.^7^ In fact, after this pandemic, the educational activities of some organizations were interrupted, and others took different approaches to carry out their educational activities.^8^ The changes made in the educational processes in the world were severe and affected the quality of education to some extent.^9,10^ Although online education has provided a solution in the current pandemic, it cannot replace offline learning, ensuring the development of the young mind for a better future. Thus, it can be understood that there is a strong need to develop effective strategies to ensure the continuity of education during future pandemics.^11,12^

 Primarily, these changes were more felt in mass education centers, so timely and effective management and control of this disease have been one of the most important concerns of these centers.^13,14^ At the world level, various strategies have been implemented to manage this disease at different levels of prevalence, and all of them have tried to control it somehow.^15,16^ Different management strategies were adopted in Iran to deal with this disease.^17^ However, the implementation and effectiveness of these strategies have been different in various organizations, and each has experienced different challenges in managing this disease. Explaining and identifying these challenges in adopting and coping strategies are very important. Based on this, the present study demonstrates the challenges of managing the COVID-19 pandemic in collective education centers in Iran.

## Materials and Methods

###  Design

 A qualitative design using a content analysis approach was used to conduct this study in some mass education centers in Iran from June to October 2022. Qualitative research helps with in-depth data analysis and provides a cultural and contextual description and interpretation of social phenomena that may not be achieved using the quantitative research tradition.^18,19^ Content analysis as a qualitative descriptive research approach is used to condense and abstract a large amount of textual data to gain new insights into the study phenomenon.^20^

###  Participants

 Participants were selected purposely and based on the following inclusion criteria: (1) Holding a managerial position in mass education centers during the COVID-19 pandemic. (2) Willingness to participate in the study and communicate their experiences. 3. Availability for the interview session. Meanwhile, people were excluded from the study under the following conditions: (1) Inability to convey their experiences effectively. (2) Refusal to answer the interviewer’s questions. (3) unwillingness to continue participation. Purposive sampling was used in this study to ensure that the participants provide sufficient data to answer the study’s primary question and fulfill its goal (Table 1). During this sampling method, it was tried to observe the maximum variation in the selection of participants. Therefore, the participants were selected from various mass education centers, which included university centers (medical sciences and basic sciences), high schools, and military training centers. In addition, collective education centers were selected from three different provinces of Iran, including Tehran, Hamadan, and Kurdistan provinces.

**Table 1:** Demographic and occupational characteristics of the participants

**ID**	**Age** **(y)**	**Gender**	**Type of training center**	**Location**	**Management ** **experience (mon)**	**Communicate**	**Data recording**
P1	39	Male	College	Kurdistan	18	Face-to-face	Voice recording
P2	43	Male	College	Tehran	21	Face-to-face	Voice recording
P3	52	Male	High school	Tehran	25	Face-to-face	Voice recording
P4	45	Female	High school	Kurdistan	16	Face-to-face	Voice recording
P5	48	Male	Military training	Tehran	14	Face-to-face	Take notes
P6	56	Male	Military training	Tehran	18	Face-to-face	Take notes
P7	48	Male	College	Kurdistan	22	Face-to-face	Voice recording
P8	54	Male	Military training	Kurdistan	26	Face-to-face	Take notes
P9	51	Male	College	Tehran	24	Face-to-face	Voice recording
P10	40	Male	College	Hamadan	21	Face-to-face	Voice recording
P11	53	Male	Military training	Kurdistan	23	Face-to-face	Voice recording
P12	51	Male	College	Hamadan	20	Face-to-face	Voice recording
P13	38	Female	High school	Hamadan	16	Face-to-face	Voice recording
P14	52	Male	College	Tehran	14	Face-to-face	Voice recording
P15	49	Male	Military training	Hamadan	22	Face-to-face	Take notes
P16	46	Female	College	Tehran	15	Face-to-face	Voice recording
P17	42	Male	College	Hamadan	21	Face-to-face	Take notes
P18	37	Female	High school	Tehran	17	Face-to-face	Voice recording
P19	52	Male	Military training	Hamadan	19	Face-to-face	Take notes

P, Participant.

###  Data collection

 From August through December 2022, there were 19 interviews with managers of mass education centers. All interviews were conducted at the participants’ workplace and only with the presence of the interviewer and participant. The interviews began with a non-structured question (tell us about your experience with COVID-19 management in this center) and progressed with semi-structured ones. Each interview lasted 35-70 minutes. Two of the interviews were repeated due to the need to clarify the obtained information. The first author (ShZ), who had prior qualitative research and interviewing experience, conducted all the interviews. 13 of the interviews were recorded with the consent of the participants, using a tape recorder. Still, six interviews were written down due to the participants’ preferences. Even though the investigation’s sample size was not predetermined, sampling continued until the data was saturated. The following analytical strategy was used to analyze the transcribed interview, and then the main themes and sub-themes were identified.

###  Data analysis

 Data analysis was carried out concurrently with data collection using the analysis method suggested by Graneheim and Lundman.^21^ The interviews were transcribed verbatim and read several times to ensure a general understanding of the participants’ statements. Next, meaning units were determined, and related codes were assigned. They were sorted and grouped into themes and subthemes by comparing constant comparison of similarities and differences between codes.^22^ The MAXQDA version 10 software (https://www.maxqda.com/qualitative-data-analysis-software) was used for data management during the analysis.

###  Rigor/trustworthiness

 Since data and findings validity were significant in qualitative research, trustworthiness criteria were utilized to validate the study.^23^ This research was based on four Lincoln and Guba’s criteria: credibility, transferability, dependability, and conformability.^24^ Prolong engagement, follow-up observations, and samplings with maximum variability were used for data credibility. To ensure the dependability of the data, the researchers were divided into two groups (ShZ, AR), and the research was conducted as two separate studies. At the same time, another expert researcher conducting qualitative research supervised the study as an external observer (RKh). Regarding conformability, the researchers avoided incorporating personal preferences into the coding process. Moreover, the codes were reviewed by the participants and two researcher colleagues (HM, FF) with the help of an independent researcher and an expert familiar with qualitative research (RKh). Data transferability was confirmed by a comprehensive description of the subject, participants, data collection, and data analysis.

## Results

 Four main themes and 11 sub-themes were developed, indicating the challenges of managing the COVID-19 pandemic in mass education centers in Iran (Table 2). The description and interpretation of the results using the participants’ direct quotations were provided as follows.

**Table 2:** The products of data analysis in this study.

**Main themes**		**Sub-themes**
1	The essence of dormitory life	The high population density in the dormitory
		Public Toilets
		Interprovincial travel
2	The inflexibility of the profession	Inapplicable health protocols
		Inflexible rules and regulations
3	Negligence	Not adhering to health protocols
		Non-acceptance of illness
		High-risk taking
4	Weakness of health-care platform	shortage of healthcare facilities
		Lack of specialized personnel
		The uni-dimensional aspect of healthcare services

###  Main theme 1: the essence of dormitory life

 Theme 1 shows that one of the most important challenges of managing COVID-19 in mass education centers in Iran has been the nature of sanatorium life. The dormitory life has provided the conditions for the virus to spread faster, making it difficult to manage the pandemic spread in these centers. This main theme consists of three sub-themes which are described in detail below.

####  The high population density in the dormitory

 The participants stated that many trainees live in each dormitory of the dormitories of mass education centers, so the population density is high. This population density and the close relationship of trainees, and the control of the disease, have provided the conditions for spreading the virus as much as possible.

*“…all these trainees live in the same hall and have a close relationship with each other. They even sleep less than one and a half meters away from each other...most of them during rest time. Some people would gather in a limited space and talk to each other, so this was the best condition for spreading the virus, which was difficult to control…” *(P5)

####  Public toilets

 In mass education centers, learners use public toilets and bathrooms, which can significantly help spread pandemic diseases.

*“...even though we disinfected the toilets and bathrooms two or three times a day during the outbreak of Corona, we did not have much control over these places when the trainees of two or three dormitories all use common toilets. That’s why one of the pollution points was these public toilets and bathrooms...” *(P16)

####  Interprovincial travel

 In mass education centers, some trainees come from different provinces of Iran. The participants stated that taking leave and making interprovincial trips greatly impacted the transfer of disease from outside to dormitories and mass education centers, a significant challenge in Corona management since most of the trainees are non-native.

*“…Corona was very rare among our trainees, but one trainee who returned from home had a cough, fever, and shiver, and a few days later, several others also got symptoms… Again many of our trainees in the dormitory were also infected…” *(P12).

###  Main theme 2: The inflexibility of the profession

 According to the analysis, one of the most important challenges in managing COVID-19 in some mass education centers is the inflexibility of the profession. In fact, due to some organizations’ sensitive and security nature, closing or adjusting the activities is impossible. For this reason, many management approaches, such as social distancing and quarantine, have not been able to be implemented in these centers. Theme 2 consists of two sub-themes, which are detailed below.

####  Inapplicable health protocols

 The participants stated that many of the standard health protocols could not be implemented in these centers because they were incompatible with the activities and nature of this profession.

*“…Well, the Ministry of Health proposed the social distancing protocol, which had acceptable results at the community level, but we could not implement this correctly... Finally, we have dormitories where 80 people are trained. They are living under the assumption that the best we could do was to reduce this number to 50 people, which is still a high density…” *(P17)

####  Inflexible rules and regulations

 The participants have stated that some professions have rules and regulations that are not very flexible because any negligence or change in this field can create a point of damage that threatens security.

*“…During this pandemic, many organizations were closed worldwide to cut the transmission chain. Our country’s organizations, such as universities, offices, and schools, were closed, or their human resources were reduced. However, this never happened in some centers because closing these centers or reducing the number of forces will be a big threat to the whole country…” *(P11)

###  Main Theme 3: Negligence

 Theme 3 expresses another management challenge of COVID-19 in mass education centers. The negligence of trainees in mass education centers has been one of the most important obstacles to managing and controlling COVID-19 in these centers, which has caused this virus to spread among people at high speed and resulted in a lack of effectiveness of health-care protocols. Negligence consists of three sub-themes which are listed below.

####  Not adhering to health protocols

 The participants stated that many people contributed to the chain of disease transmission by not following health protocols during this disease. These people were one of the most important obstacles to disease management.

*“… we had programs where we fully explained the importance of following health protocols to the trainees and provided them with the necessary facilities such as masks and hand sanitizer. Unfortunately, some did not comply with the protocol. They didn’t follow the rules, which made our protocols ineffective, and the negligence of these people spread the disease more…” *(P13)

####  Non-acceptance of illness

 The participants have stated that one of the most important challenges in managing this global disease in mass education centers is the trainees’ lack of acceptance of the disease. People during this period did not consider this virus a dangerous disease and believed that all the symptoms were simply colds or cases of flu.

*“… One of our most important problems was that we could not justify to some people that this is a new and dangerous disease because when these people had symptoms, they believed that they had a cold and it is not a dangerous pathogen... Even some of these people resisted receiving medicine and said that we did not take medicine during previous colds and got better by ourselves…” *(P4)

####  High-risk taking 

 Based on the participants’ demonstrations, it can be understood that some trainees’ high-risk taking has been an important obstacle to controlling this disease in mass education centers. Due to such phenomena as pride and misplaced self-confidence, some people have taken a high risk regarding the disease and believed their body is not vulnerable to it.

*“…among our trainees, there were people who believed that their bodies were invulnerable to such a simple disease... for example, they said that because we are athletes, our bodies have the necessary preparation to deal with this. Or our disease is not supposed to be acute, which is why many of them did not go to medical centers despite the symptoms or resisted receiving care services, which caused many to enter the acute phase of the disease, which became a big problem for us…” *(P15)

###  Main theme 4: Weakness of health-care platform

 Theme 4 expresses one of the most important challenges of COVID-19 in mass education centers in Iran. This theme shows that the weakness of the health-care platform has caused mass education centers to experience many problems in managing this pandemic. The dimensions of liability in this field have been different, which are mentioned as sub-themes below.

####  Shortage of healthcare facilities

 The participants have stated that one of the most critical challenges in managing this pandemic is the lack of healthcare facilities in these centers. This lack includes the quantity and quality of facilities and equipment needed to address an outbreak.

*“…The onset of this disease in our center triggered many problems in the field of facilities, each of which made it difficult to manage this disease in some way. For example, we had a severe shortage of masks and disinfectants for staff and trainees at the beginning of this disease. In the later stages, the defect and incompatibility of the medical center were evident. It could not respond to this volume of respiratory patients, so we had to transfer some people to other medical centers…” *(P14)

####  Lack of specialized personnel

 The participants’ experiences show that the lack of specialized staff in this field is another obstacle to managing COVID-19 in mass education centers. The shortage of technical personnel includes all people involved in healthcare, especially doctors specializing in infectious and respiratory diseases, nurses with experience in the respiratory special care department, and epidemiologists living in these centers.

*«…It is always ideal that in mass centers, most human needs are met inside the center, so there is no need to go outside the center to meet the needs such as care and treatment. Thus, these things are significant in terms of safety tactics. However, with the spread of COVID-19 among trainees, as we did not have enough medical personnel, especially lung and respiratory disease specialists, it was sometimes necessary to send our staff to medical centers outside the headquarters…» *(P19)

####  The uni-dimensional aspect of healthcare services

 The participants have stated that with the spread of this disease, sometimes the health care services in these centers have become uni-dimensional so that most health care attention has been focused on this disease and other routine needs have been neglected.

*“… Before the Corona outbreak, we provided students a wide range of healthcare services. For example, our mental health consultations were more extensive, oral and dental services were performed better, and skin disease screenings were more accurate. However, as the disease progressed and, in particular, as new peaks emerged, their significance diminished. Unfortunately, given our limited resources, we had to focus more on protecting the personnel from Corona and less on other issues…” *(P9)

## Discussion

 This study aimed to explain the challenges of managing the COVID-19 pandemic in mass education centers in Iran. As a result of this study identified many factors as challenges, each of which has been an obstacle to controlling and managing the COVID-19 pandemic in mass education centers. As a result of the analysis, the challenges of managing the COVID-19 pandemic in these centers have been in the form of four main themes: the nature of dormitory life, the nature of inflexibility of the profession, negligence, and the weakness of the healthcare platform, each of which has several sub-themes.

 As a challenge to managing the COVID-19 pandemic in mass education centers in Iran, the nature of dormitory life refers to factors that naturally exist in a dormitory and facilitate disease spreading. In this context, several previous studies have shown that living in communal dormitories provides the basis for transmitting various types of infection, and many people get infected in that environment.^25-27^ According to this study’s results, one of dormitory life’s problems is population density, which has been a major challenge in managing the COVID-19 pandemic in mass education centers in Iran. Regarding the availability of infection transmission in dormitories, Kak has stated that there is a large group of people in closed spaces, such as cruise ships, military barracks, and university dormitories, often associated with an increased risk of certain infections.^27^ In our study, it was demonstrated that the frequent use of public toilets by people is another issue that has made it difficult to manage the COVID-19 disease in the centers; Dancer et al stated that using public toilets is a potential risk for the spread of COVID-19 that cannot be ignored.^28^ Another challenge in managing the COVID-19 pandemic in mass education centers in Iran has been the interprovincial commuting of trainees living in these centers, which is a risk factor that can significantly increase the number of cases of COVID-19 as shown in the study of Ahmadi et al.^29^

 The nature of the inflexibility of the profession as the second central theme obtained refers to the inherent conditions of some mass education centers in Iran, which do not allow flexibility and adaptation to the acute conditions of the pandemic. This inflexibility has been more understandable in mass education centers, which have a security nature. Many organizations and professions have used new approaches to adapt to the new conditions. To reduce disease transmission, they have adjusted their activities so that people have minimal physical contact with each other.^30,31^ However, in jobs such as the military jobs, it is impossible to shut down activities. Even in many countries, military forces have helped the health-treatment systems in the fight against the Corona pandemic.^32-34^

 Another main theme of this study is negligence, which means the lack of sensitivity and taking necessary self-care measures against COVID-19 among trainees in military training centers. According to the officials of these centers, one of the most important challenges in managing the COVID-19 pandemic has been defined mainly as non-adherence to health protocols, patients’ non-acceptance of the disease, and their high risk-taking. In this regard, Choudhary et al have stated that the negligence of the general public can facilitate the further spread of the COVID-19 pandemic and make the management of this pandemic difficult.^35^ Also, in this context, de Micy Ferreira Cintra et al have stated that not following health protocols and especially not using masks in public centers is like the calm before the storm and will result in heavy waves of coronavirus.^36^

 Another critical challenge of mass education centers in managing the COVID-19 pandemic in these centers is the weakness of the healthcare platform, which consists of three sub-themes, each of which is considered a fundamental challenge in some way. According to the statements of the officials of mass education centers in Iran, factors such as the lack of healthcare facilities, the lack of specialized human resources, and the one-dimensionality of healthcare services have been destructive factors that have made the management of this COVID-19 pandemic in these centers difficult. Various studies have shown that one of the most important factors in managing the COVID-19 pandemic is the availability of necessary healthcare facilities. The shortage of health-care beds makes it challenging to control this disease in many countries.^37,38^

 Some limitations need to be acknowledged in this study. It outlines the COVID-19 management challenges and does not provide information on how to address them, which will be the subject of future research. Secondly, the challenges identified have been based on the statements of the managers of the mass education centers, which may have been biased in expressing their experiences. This is one of the inherent limitations of qualitative studies.

## Conclusion

 The results of this study show that there have been many challenges in managing the COVID-19 pandemic in mass education centers in Iran, making it difficult to control this pandemic correctly and timely. The variety of challenges identified in this research is wide. However, they can be categorized into four major themes. The themes include the nature of dormitory life, the inflexible nature of the profession, negligence, and the weakness of the healthcare platform, each of which has several sub-themes. Based on these findings, it can be said that the challenges experienced in these centers were not one-dimensional. Many factors affected the management of this pandemic, including the inherent characteristics of the organization, the available facilities, and how people deal with this pandemic. Therefore, it is recommended that the studied centers prepare in advance for future pandemics so that (1) inflexible centers can develop patterns compatible with their conditions and (2) mass education training centers can encourage trainees to take care of themselves and seek alternative care. (3) Mass education centers should enhance their healthcare platforms so that they do not face management challenges in the event of future pandemics. The findings of this study illuminate the challenges experienced in COVID-19 management in Iranian mass education centers so that healthcare administrators can apply these findings to strengthen their organizations in the event of a new pandemic. Also, these findings can help future studies consider interventions to address these challenges and design adaptable management patterns for pandemic management in mass education centers.

## Acknowledgments

 The authors would like to thank all participants who shared their valuable experiences in this study. We are also grateful to Dr Robabe Khalili, School of Nursing and Midwifery, Baqiyatallah University of Medical Sciences, Tehran, Iran, for external supervision in conducting this study.

## Competing Interests

 The author(s) declared no potential conflicts of interest concerning this article’s research, authorship, and/or publication.

## Ethical Approval

 The present study was approved under the ethical code number IR.BMSU.REC.1400.169 from Baqiyatallah University of Medical Sciences in 2022. Before starting the interview, the researcher introduced herself and provided the necessary information to the participant regarding her expertise, job position, and research interests. The purpose of the study was explained, and all participants’ consent was obtained. All participants were informed that the obtained information would be kept private and that no personal information would be shared.

## Funding

 The study was financially supported by Baqiyatallah University of Medical Sciences.
